# Detection of Antibiotic‐Resistant *Campylobacter* on Retail Chicken in New Zealand: A Sentinel Survey

**DOI:** 10.1002/snz2.70066

**Published:** 2026-07-30

**Authors:** Kathleen J. Sircombe, Daniel Pletzer, Sarah Hook

**Affiliations:** ^1^ Department of Microbiology and Immunology Faculty of Biomedical Sciences University of Otago Dunedin New Zealand; ^2^ School of Pharmacy University of Otago Dunedin New Zealand

**Keywords:** AMR, antimicrobial resistance, *Campylobacter jejuni*, foodborne pathogens, foodborne transmission

## Abstract

Antibiotics are relied upon to treat and prevent infections in humans, animals, and plants. However, antimicrobial use, particularly when unnecessary or inappropriate, has contributed to the emergence and dissemination of antimicrobial resistance (AMR). Foodborne transmission of antibiotic‐resistant bacteria is an increasing public health concern, particularly through poultry contaminated with *Campylobacter jejuni*. In this study, the prevalence of *Campylobacter* contamination and associated antimicrobial resistance in fresh retail chicken purchased around New Zealand was investigated. *Campylobacter* was detected in 50% (12/24; 95% CI: 31.3%–68.7%) of samples using selective culture, PCR, and sequencing. Among confirmed isolates, high levels of resistance were observed to tetracycline (92%) and ciprofloxacin (83%), while 83% were susceptible to erythromycin. No isolate was fully susceptible to all interpreted antibiotics. These preliminary findings indicate that antimicrobial‐resistant *Campylobacter* can be detected in fresh retail chicken in New Zealand and support the need for larger, probabilistic surveillance studies to generate national prevalence and resistance estimates. Continued surveillance, stricter policies on contaminated poultry products, and development of alternatives to traditional antibiotics are recommended to protect public health.

## 1 Introduction

The World Health Organization has declared antimicrobial resistance (AMR) as one of the top 10 global public health threats, due to increasing treatment failures and the rapid global spread of multidrug‐resistant pathogens (World Health Organization 2015, 2020). Many factors contribute to the growing incidence of AMR, including the overuse of antimicrobials in humans and animals, which creates strong selective pressure for resistance (Xie et al. 2018). The use of antibiotics in agriculture has come under international scrutiny, with many governments implementing regulations restricting their use (European Food Safety Authority 2022). Although antibiotics cannot be used for growth promotion in New Zealand (NZ) (Hillerton et al. 2017), in 2022, around 55% of total antibiotics sold were registered for use in animal feeds, with sales per species being highest in pigs and poultry. Historically, antibiotics registered for administration in poultry feed contained zinc bacitracin (86%) (Ministry of Agriculture and Forestry 2011; Hillerton et al. 2021). In 2023, NZ phased out the use of bacitracin in poultry feed, reserving antibiotics for the treatment of active infections (MPI 2025). The NZ Veterinary Association has set a goal for the country's agriculture to be antibiotic‐free, except in emergency cases, by 2030. NZ Food Safety and various industry partners have implemented initiatives to reduce animal antibiotic use and mitigate the development of AMR (van den Bogaard and Stobberingh 2000; New Zealand Veterinary Association 2018). These initiatives align with a One Health approach linking human, animal, and environmental health.

Campylobacteriosis, a gastrointestinal illness caused by *Campylobacter* species, is a major cause of foodborne illness globally (Igwaran and Okoh 2019). In NZ, an estimated 500,000 cases were reported between 2009 and 2018, resulting in 284 deaths and approximately US$380 million in economic loss (Baker et al. 2021). Annual notifications remain high, with under‐reporting suggesting true incidence is 10–20 times higher (Horn 2023). The majority of campylobacteriosis in New Zealand is caused by *Campylobacter jejuni* and, to a lesser extent, *C. coli*. Seasonal peaks have been observed, with infection rates doubling across the summer months. Approximately two‐thirds of campylobacteriosis cases in NZ are associated with ingestion of contaminated food, typically undercooked poultry or cross‐contamination during preparation. To reduce foodborne illness, New Zealand Food Safety Authority (NZFSA) introduced a mandatory *Campylobacter* Performance Test (CPT) in 2008, resulting in a 58% decrease in campylobacteriosis notifications the following year (Sears et al. 2011). Since then, case numbers have remained stable (Baker et al. 2025), even with progressive tightening of *Campylobacter* limits in 2013 and 2016 (van der Logt et al. 2018).

Therefore, this study aimed to assess the prevalence of *Campylobacter* contamination in fresh retail chicken from major cities in New Zealand and to characterize the antimicrobial susceptibility of confirmed isolates to clinically relevant antibiotics.

## 2 Materials and Methods

### 2.1 Collection of Retail Chicken Samples

This study used a pragmatic, sentinel sampling approach. Twelve prepacked fresh skinless chicken breasts and 12 skin‐on chicken drumsticks (*n* = 24 total samples) were collected in May 2021, with a balanced plan of 3 breasts and 3 drumsticks per city (Auckland, Wellington, Christchurch, and Dunedin) from two different supermarkets and one butcher. Due to the small sample size and risk of identifying individual producers, manufacturer‐level findings were not analyzed or reported. No formal a priori statistical power calculation was performed because the study aimed to generate preliminary, geographically distributed data rather than to provide definitive, population‐level prevalence estimates. Samples were selected based on laboratory throughput, time, and funding constraints.

On the day of purchase, samples were transported to the laboratory in Dunedin, inside a cooler bag with ice packs and an Emerson G PDF Mini USB temperature monitor to measure the internal cooler bag temperature.

### 2.2 Sampling and Culture of Retail Chicken Samples

The outer packaging was sprayed with 70% ethanol and partially removed. Triplicate samples of chicken (approximately 5 cm^3^, weighing 10 g) were removed and placed in 5 mL of enrichment media (Nutrient Broth II, 5% lysed horse blood, Thermo Fisher) supplemented with Oxoid Preston Campylobacter selective media (Thermo Fisher) and Oxoid Campylobacter growth supplement (Thermo Fisher) in a 20 mL sterile tube and vortexed for 30 s. The chicken was removed, and 10 mL of media was added before incubation at 42 °C for 48 h under microaerophilic conditions with an Oxoid CampyGen 2.5L sachet (Thermo Fisher‐CN0025A). Included as controls were blank enrichment media and three dominant *Campylobacter* strains in New Zealand sourced from The Institute of Environmental Science and Research ESR (now New Zealand Institute for Public Health and Forensic Science; PHF) as positive controls: *C. jejuni* (ATCC 33291), *C. coli* (ATCC 33559), and *C. upsaliensis* (ATCC 43954).

Following selective enrichment, samples were then serially diluted in phosphate‐buffered saline (PBS, pH 7.4) and plated onto Campylobacter selective agar plates (Campylobacter agar base, 5% lysed horse blood, supplemented with Campylobacter selective media and Campylobacter growth supplement, Thermo Fisher). Plates were incubated at 42 °C for 48 h under microaerophilic conditions and examined for bacterial growth. Colony counts were normalized to the mass of the individual chicken subsample processed. Because enumeration was performed after selective enrichment, these values should be interpreted as post‐enrichment semiquantitative counts rather than direct estimates of the initial bacterial load on the retail product. In the event of no or insufficient bacterial growth, plates were re‐incubated under the same conditions for a further 48 h. Any sample that had *Campylobacter* on at least one of the triplicate samples was classified as positive. Glycerol stocks were made from individual colonies picked from Campylobacter selective agar plates and grown overnight at 42 °C in enrichment media under microaerophilic conditions with an Oxoid CampyGen 2.5L sachet. These were then frozen in 12.5% Glycerol at 80 °C.

### 2.3 *Campylobacter* spp. Detection Method

Glycerol stocks of samples were streaked onto Campylobacter selective agar plates and incubated at 42 °C overnight in microaerophilic conditions. Bacteria were scraped from plates, resuspended in PBS, and then centrifuged at 8,000 RPM for 3 min. DNA was extracted from bacterial pellets using NucleoSpin DNA extraction kits (Macherey‐Nagel), and the quantity and quality of DNA assessed using a NanoDrop (Thermo Fisher).

Gradient Polymerase Chain Reaction (PCR) was utilized to establish the optimal annealing temperatures for the amplification of genes specific for *C. jejuni*, *C. coli*, and *C. upsaliensis*, as well as for *C. jejuni* 23S RNA as a positive control (Table S2). Primers based on published literature were tested at various annealing temperatures and concentrations to determine optimal PCR conditions for amplification. PCR was performed on 50 ng of DNA in a Bio‐Rad T100 thermal cycler (Table S3), and products were visualized on a SYBRsafe (Applied Biosystems) stained 1.5% agarose gel alongside a 100 bp DNA ladder (Invitrogen) Samples were subjected to Sanger sequencing (Genetic Analysis Service at the University of Otago) and analyzed using the National Centre for Biotechnology Information (NCBI) Basic Local Alignment Search Tool (BLAST) tool.

### 2.4 Antibiotic Susceptibility Assay

Cultures taken from glycerol stocks were inoculated into Nutrient broth II and incubated under microaerophilic conditions at 42 °C for 24 h. After incubation, samples were adjusted to McFarland turbidity 0.5, and 100 µL was spread onto Campylobacter selective plates (McFarland 1907). Oxoid Antibiotic discs (Thermo Scientific) containing chloramphenicol (30 µg), ciprofloxacin (5 µg), erythromycin (15 µg), and tetracycline (30 µg) were placed onto each plate, followed by incubation in microaerophilic conditions at 42 °C for a further 24 h. Zone‐of‐inhibition (ZOI) diameters were measured in millimeters after incubation. For each antimicrobial disk, at least three diameters were measured across different orientations, and the median diameter was recorded and used for interpretation. The antimicrobial panel was selected to include agents of clinical and surveillance relevance for *Campylobacter*, while retaining partial comparability with previous New Zealand poultry studies. Ciprofloxacin and erythromycin were included because they are relevant to the treatment of severe human campylobacteriosis, and tetracycline was included because tetracycline resistance is commonly monitored in *Campylobacter* AMR surveillance. Chloramphenicol was included for historical comparability with previous New Zealand poultry data, although breakpoints were not determined because no resources were available at the time of the study. European Committee on Antimicrobial Susceptibility Testing (EUCAST) Clinical Breakpoints (version 16.0) and Clinical & Laboratory Standards Institute (CLSI) M45 for *Campylobacter* were used to determine whether an isolate was susceptible or resistant to ciprofloxacin, tetracycline, and erythromycin (Clinical and Laboratory Standards Institute 2015; The European Committee on Antimicrobial Susceptibility Testing 2026). Bacterial samples were deemed resistant if any of the three replicates were scored as resistant.

### 2.5 Statistical Analysis

Statistical analyses were performed using Fisher's exact test to compare proportions between groups (location, product type, and retail source). Wilson/Brown 95% confidence intervals were calculated for prevalence estimates. A *p*‐value <0.05 was considered statistically significant. Analyses were conducted in Prism (version 10.6.1).

## 3 Results

### 3.1 Prevalence of *Campylobacter* spp. in Retail Chicken

**FIGURE 1:**
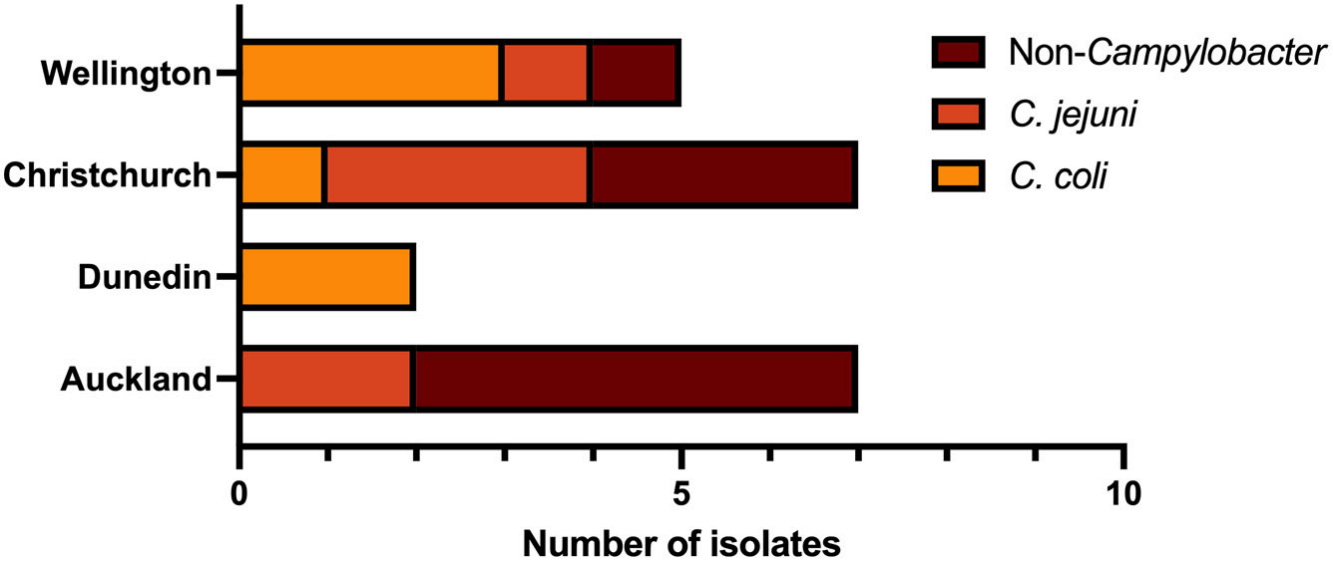
Species identification of presumptive *Campylobacter* isolates by PCR and Sanger sequencing. Presumptive *Campylobacter* spp. were subjected to PCR amplification followed by sequencing to resolve species‐level identity. Samples were classified as *C. coli*, *C. jejuni*, or non‐*Campylobacter* (such as *Lactococcus lactis*). A subset of samples yielded mixed results, testing positive for *Campylobacter* spp. and *L. lactis*.

Fresh processed chicken samples (*n* = 24; 12 skinless breast and 12 skin‐on drumsticks) were purchased from supermarkets and butchers in four major New Zealand cities (Figure 1). All samples were transported under chilled conditions (average <7 °C; Table S1), although temperature monitoring indicated that the Auckland samples exceeded 7 °C for approximately 30 min during transit. This may have affected bacterial load but would not have impacted the presence/absence determinations; therefore, these samples were included in the study.

Seventeen of the 24 chicken samples (70.8%) produced growth on Campylobacter selective media (Figure 1, Table 1). Auckland had the highest proportion of potential *Campylobacter* (100%), followed by Wellington (83.3%), Christchurch (66.7%), and Dunedin (33.3%). However, these differences were not statistically significant (Fisher's exact test, *p* = 0.54). Semiquantitative colony counts, normalized to the original chicken subsample, ranged from 10^1^ to 10^6^ CFU/g, with the majority containing between 10^4^ to 10^5^ CFU/g (Figure S1).

**TABLE 1:** Number of presumptive and confirmed *Campylobacter*‐positive retail chicken samples, by location (*n* = 6; 3 breast and 3 drumstick samples).

Location	No. presumptive positive by agar selection (%)	No. positive by PCR (%)	No. positive by PCR and/or sequencing (%)
Auckland	6 (100)	2 (33.3)	2 (33.3)
Wellington	5 (83.3)	4 (66.7)	4 (66.7)
Christchurch	4 (66.7)	1 (16.7)	4 (66.7)
Dunedin	2 (33.3)	2 (33.3)	2 (33.3)
Total	17 (70.8)	9 (37.5)	12 (50)
95% CI (lower–upper)	50.83%–85.09%	21.16%–57.29%	31.42%–68.57%

To confirm the presence and identity of *Campylobacter*, a multiplex PCR assay targeting species‐specific genes was developed and optimized. Nine samples were identified as being *C. jejuni*, *C. coli* or *C. upsaliensis* (Table 1). The remaining eight PCR‐negative samples were subjected to Sanger sequencing of 23S rRNA amplicons, which identified three additional *C. jejuni* isolates and five *Lactococcus* spp. isolates. Overall, this yielded a confirmed *Campylobacter* contamination rate of 50% (12/24 samples; 95% CI: 31.3%–68.7%).

### 3.2 Antimicrobial Susceptibility of *Campylobacter* from Retail Chicken

Confirmed *Campylobacter* isolates (*n* = 12) were assessed for susceptibility to three antibiotics using EUCAST Clinical Breakpoints and CLSI guidelines (Clinical and Laboratory Standards Institute 2015; The European Committee on Antimicrobial Susceptibility Testing 2026) (Tables 2 and S4). Chloramphenicol ZOI diameters were recorded descriptively only, because no validated *Campylobacter*‐specific interpretive criterion was available. 10/12 (83%) of isolates were susceptible to erythromycin. In contrast, resistance to ciprofloxacin, and tetracycline was widespread (Table 2). No isolate was susceptible to all antibiotics tested. Comparisons of resistance prevalence across the North versus the South island revealed no statistically significant differences for ciprofloxacin or tetracycline resistance (Fisher's exact test, both *p =* 1.0). Similarly, resistance did not differ significantly between skinless breast and skin‐on drumstick samples for ciprofloxacin (*p* = 0.45) or tetracycline (*p =* 1.0). Multidrug resistance, defined as resistance to ≥2 tested antibiotics, was observed across product types, but this difference was not statistically significant (*p* = 0.18).

**TABLE 2:** Proportion of isolates (*n* = 12) demonstrating antibiotic resistance as determined using EUCAST Clinical Breakpoints (The European Committee on Antimicrobial Susceptibility Testing 2026).

	Ciprofloxacin	Erythromycin	Tetracycline
Auckland	2/2	0/2	1/2
Wellington	3/4	0/4	4/4
Christchurch	3/4	1/4	4/4
Dunedin	2/2	1/2	2/2
Total	10/12 (83.3%)	2/12 (16.7%)	11/12 (91.7%)
95% CI (lower–upper)^a^	55.20%–95.30%	4.70%–44.80%	64.61%–98.51%

a Based on Wilson/Brown 95% CI.

## 4 Discussion

This study provides a preliminary sentinel snapshot of *Campylobacter* contamination and antimicrobial resistance in fresh retail chicken (*n* = 24 products) purchased across four major cities in New Zealand. Because of the small sample size and single time‐point collection, these data cannot provide reliable national estimates. *Campylobacter* spp. were confirmed in 50% of sampled products (12/24; 95% CI 31.4%–68.6%), and concerning levels of resistance to tetracycline (11/12; 91.7%) and ciprofloxacin (10/12; 83.3%) were frequently observed among confirmed isolates. These findings indicate that retail poultry remains a relevant source of potential exposure to antimicrobial‐resistant *Campylobacter*. Our confirmed *Campylobacter* prevalence appeared lower than earlier New Zealand retail chicken surveys reporting prevalences of 89.1% in 2003–2004 (230 retail chicken items) and 69.7% in 2009 (175 retail chicken items) (Wong et al. 2007; Wong and Hudson 2011). This apparent reduction may be consistent with the impact of New Zealand's poultry‐focused *Campylobacter* control measures, including the introduction of mandatory *Campylobacter* performance targets in 2008 and subsequent tightening of regulatory limits. However, direct comparisons between studies should be made with caution. The present study used a small pragmatic sample size, was conducted at a single time point, and included only selected fresh retail chicken products, whereas earlier surveys used larger sample sizes and may have differed in sampling frame, product type, season, and microbiological methods. Consequently, the presented study was not powered to detect small differences in *Campylobacter* presence, and the lack of statistically significant differences should not be interpreted as definitive evidence of no actual difference. To help readers interpret uncertainty, we present Wilson/Brown 95% confidence intervals for all proportions and report raw counts alongside percentages. Future work should use probabilistic sampling with formal sample‐size calculations, sample over multiple time points, and include explicit sampling across manufacturers and processing plants to permit robust prevalence estimates and trend analyses.

The emergence of resistance to tetracycline and fluoroquinolones in the present study contrasts with retrospective analyses from 2006, which found that nearly all *Campylobacter* isolates from poultry carcasses were fully susceptible (Pleydell et al. 2010). Similar increases in resistance have been reported internationally, including in Korea (Kim et al. 2016; Gahamanyi et al. 2020), Malaysia (Teh et al. 2019; Sabsabi et al. 2021), and Australia (Abraham et al. 2020), although absolute resistance frequencies vary between countries and depend on antimicrobial usage policies. In New Zealand, available evidence suggests that substantial quinolone/tetracycline resistance among poultry‐associated *Campylobacter* was not prominent in the mid‐2000s but was evident by the mid‐2010s, including through the detection of fluoroquinolone‐ and tetracycline‐resistant lineages (Pleydell et al. 2010), such as sequence type ST6964, in both poultry and human clinical isolates (French et al. 2019).

Cornelius et al. (2024) also observed resistance (16.6%) to quinolones and tetracycline among *Campylobacter* spp. isolated from New Zealand poultry. In their multi‐year study, resistance to quinolones/tetracycline was widespread and increasing, whereas resistance to erythromycin and gentamicin remained zero. Although the resistance frequency reported by Cornelius et al., was lower than the proportions observed in the present small retail survey, both studies indicate the persistence of resistance to clinically relevant antimicrobials among poultry‐associated *Campylobacter* in New Zealand. The presence of resistant isolates in retail chicken, therefore, aligns with the ongoing concern that poultry remains an important reservoir for resistant *Campylobacter*.

However, differences in sample size and analytical methodology prevent direct comparison between studies or precise identification of the time point at which resistance increased. Because fluoroquinolones are frontline agents for managing severe campylobacteriosis, increasing resistance among foodborne isolates poses a threat to effective treatment and may prolong illness. Importantly, the high frequency of ciprofloxacin resistance observed in this small dataset should not be interpreted as evidence of fluoroquinolone overuse in New Zealand poultry. Fluoroquinolones are not registered for use in poultry in New Zealand, and previous genomic work has suggested that fluoroquinolone‐resistant *C. jejuni* ST6964 in New Zealand poultry is unlikely to have arisen through direct fluoroquinolone use in the domestic poultry food chain. Instead, the presence of fluoroquinolone‐resistant *Campylobacter* may reflect the introduction and clonal expansion of resistant lineages, co‐selection with resistance to other antimicrobials, historical or indirect selection pressures, or environmental and other reservoirs. The present study cannot distinguish between these mechanisms, and larger longitudinal studies integrating antimicrobial use, genomic surveillance, and retail sampling would be needed to do so.

National initiatives such as the “*Campylobacter* Action Plan” and the “New Zealand Antimicrobial Resistance Action Plan” continue to drive reductions in foodborne campylobacteriosis and promote the responsible use of antibiotics in primary production (McNeill et al. 2017; New Zealand Food Safety 2020). While these efforts have contributed to declining contamination rates, our data indicate that resistance to frontline antimicrobials remains common in retail poultry products. The policy implications of these findings should be considered within the context of New Zealand's existing food safety framework. Prior studies have highlighted low consumer awareness of *Campylobacter* contamination on raw poultry and proposed mandatory warning labels as a potential risk‐mitigation strategy (Allan et al. 2018). While warning labels may reinforce risk at the point of sale, comprehensive public education campaigns focused on safe food preparation are likely to be more effective in preventing campylobacteriosis. Other potential regulatory actions include tightening performance targets for *Campylobacter* on carcasses during processing and/or restricting the sale of fresh chicken that exceeds agreed contamination thresholds. Freezing has been shown to significantly reduce *Campylobacter* load, and some jurisdictions preferentially encourage consumers to purchase frozen poultry as a risk‐mitigation strategy. Further refinement of industry controls, strengthened consumer education, and the development of alternatives to traditional antimicrobial use, such as targeted or triggerable antibiotic prodrugs (Ross et al. 2024) or antimicrobial peptides (Kabiraz et al. 2026), may help reduce selective antibiotic pressure while maintaining animal welfare.

In conclusion, retail chicken in New Zealand continues to serve as a potential vehicle for transmission of antimicrobial‐resistant *Campylobacter*. Ongoing surveillance and coordinated sector responses will be essential to limit the spread of resistant Campylobacter and protect public health.

## Funding

This study was supported by an Otago Medical Research Foundation Laurenson Grant awarded to SH (LA‐393). KJS was supported by a University of Otago Doctoral Scholarship throughout her PhD study.

## Conflicts of Interest

The authors declare no conflicts of interest.

## Supporting information


**Figure S1:** Average bacterial count by location. Number of contaminating *Campylobacter* bacteria (CFU/g). Error bars show the geometric mean with geometric standard deviation.
**Table S1:** Temperature logger data during transportation of chicken samples. Aside from the Auckland samples, all the samples maintained temperatures of less than 8 °C during transportation.
**Table S2:** Primer pair sequences for target genes (1).
**Table S3:** Multiplex PCR protocol. All primer stocks are 100 µM.
**Table S4:** AMR Zone of Inhibition (ZOI) data. ZOI is shown in mm.

## Data Availability

All data is available in the Supporting information, or by contacting the corresponding authors, Sarah Hook (sarah.hook@otago.ac.nz) or Daniel Pletzer (daniel.pletzer@otago.ac.nz).
