# Supplementary material for: Detection of Antibiotic‐Resistant Campylobacter on Retail Chicken in New Zealand: A Sentinel Survey
Source: J R Soc N Z. 2026 Jul 30;56(4):e70066. doi: 10.1002/snz2.70066 (PMC13422752; doi:10.1002/snz2.70066)
Supplement: Supplementary file 1 — Figure S1: Average bacterial count by location. Number of contaminating Campylobacter bacteria (CFU/g). Error bars show the geometric mean with geometric standard deviation. Table S1: Temperature logger data during transportation of chicken samples. Aside from the Auckland samples, all the samples maintained temperatures of less than 8 °C during transportation. Table S2: Primer pair sequences for target genes (1). Table S3: Multiplex PCR protocol. All primer stocks are 100 µM. Table S4: AMR Zone of Inhibition (ZOI) data. ZOI is shown in mm. [file SNZ2-56-e70066-s001.pdf]

Detection of antibiotic-resistant *Campylobacter* on retail chicken in New Zealand: a sentinel survey

Kathleen J Sircombe[1,2], Daniel Pletzer[1*], and Sarah Hook[2*]

[1]Department of Microbiology and Immunology, School of Biomedical Sciences, University of Otago, 9054, Dunedin, New Zealand

[2]School of Pharmacy, University of Otago, 9054, Dunedin, New Zealand

*Corresponding author(s): sarah.hook@otago.ac.nz, daniel.pletzer@otago.ac.nz

Supplementary Information

SI Table S1: Temperature logger data during transportation of chicken samples. Aside from the Auckland samples, all the samples maintained temperatures of less than 8 ℃ during transportation.

Location	Lowest temperature (℃)	Highest temperature (℃)	Average temperature (℃)
Auckland	3.3	10.2	6.69
Wellington	-0.4	0.2	-0.1
Christchurch	4.3	5.3	4.65
Dunedin	3.2	6.5	3.59

SI Table S2: Primer pair sequences for target genes (1).

Target gene	Primer	Identifier gene length (bp)	Primer sequence (5'-3')	GenBank accession number
C. jejuni hipO	CJF	323	ACTTCTTTATTGCTTGCTGC	Z36940
	CJR		GCCACAACAAGTAAAGAAGC	
C. coli glyA	CCF	126	GTAAAACCAAAGCTTATCGTG	AF136494
	CCR		TCCAGCAATGTGTGCAATG	
C. upsaliensis glyA	CUF	204	AATTGAAACTCTTGCTATCC	AF136496
	CUR		TCATACATTTTACCCGAGCT	
C. jejuni 23S rRNA (for all *23S* analysis)	23SF	650	TATACCGGTAAGGAGTGCTGGAG	Z29326
	23SR		ATCAATTAACCTTCGAGCACCG	

SI Table S3: Multiplex PCR protocol. All primer stocks are 100 µM.

PCR component	Volume for one reaction (µL)
DNA	50 ng
F primers *C. jejuni*	1
R primers *C. jejuni*	1
F primers *C. coli*	1
R primers *C. coli*	1
F primers *C. upsaliensis*	2
R primers *C. upsaliensis*	2
F primers *C. jejuni 23S*	0.3
R primers *C. jejuni 23S*	0.3
dNTP mix (Invitrogen)	0.4
5 × SuperFi II Buffer (Invitrogen)	4
Platinum SuperFi II DNA Polymerase (Invitrogen)	0.4
$MgCl_2$	0.1
Water	To 20uL

SI Table S4: AMR Zone of Inhibition (ZOI) data. ZOI is shown in mm.

Sample	Chloramphenicol	Ciprofloxacin (R < 26)[1]	Erythromycin (R < 20)	Tetracycline (R < 30)
C. jejuni	32	40	32	24*
C. coli	31	39	31	21*
C. upsaliensis	32	38	30	22*
C. lari	27	27	28	19*
**Auckland**				
**DS 1**	33	20*	35	31
**DS 2**	37	19*	38	22*
**Wellington**				
**DS 1**	30	20*	30	21*
**DS 2**	29	32	30	21*
**SB 1**	28	19*	28	19*
**SB 2**	29	22*	30	20*
**Christchurch**				
**DS 1**	44	41	40	23*
**DS 2**	27	18*	32	24*
**SB 1**	32	17*	12*	16*
**SB 2**	30	22*	34	20*
**Dunedin**				
**SB 1**	34	14*	36	24*
**SB 2**	29	16*	14*	19*

[1] Values in brackets taken from EUCAST Clinical Breakpoint Table v. 16.0 (2).
[*] Indicates that the sample is considered resistant when measured by the appropriate EUCAST guidelines.

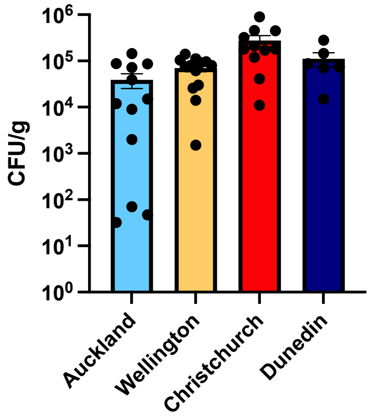

SI Figure S1: Average bacterial count by location. Number of contaminating *Campylobacter* bacteria (CFU/g). Error bars show the geometric mean with geometric standard deviation.

References:

1) Wang G, Clark CG, Taylor TM, Pucknell C, Barton C, Price L, Woodward DL, Rodgers FG. Colony multiplex PCR assay for identification and differentiation of *Campylobacter jejuni, C. coli, C. lari, C. upsaliensis*, and *C. fetus* subsp. fetus. J Clin Microbiol. 2002 Dec;40(12):4744-7. doi: 10.1128/JCM.40.12.4744-4747.2002.

2) The European Committee on Antimicrobial Susceptibility Testing. *EUCAST Disk Diffusion Test Methodology – Clinical breakpoints (v 16.0)*. 2026. https://www.eucast.org/bacteria/clinical-breakpoints-and-interpretation/clinical-breakpoint-tables/ (accessed June 2026).
